# Proceedings of the Annual Convention of the Connecticut Medical Society; Transactions of the Medical Society of the State of New York

**Published:** 1848-09

**Authors:** 


*Proceedings of the Annual Convention of the Connecticut Medical Society, May,* 1848, *together with a list of Members, and the Annual Address.* Hartford. 1848.

*Transactions of the Medical Society of the State of New York.* Vol. vii. part 2. Albany, 1848.

The Convention of the State Medical Society of Connecticut, is constituted of the members or delegates from the county societies of the State, eight in number, containing altogether three hundred and ninety-six members.

The annual address of the present year was delivered by B. Fordyce Barker, M. D. It is entitled "Remarks on some forms of disease of the Cervix Uteri," and contains some good practical suggestions.

The *Transactions* of the New York State Medical Society are always interesting, as the meetings of so large a body of profes sional gentlemen must ever be. The present publication, be sides the usual abstract of proceedings and reports, contains addresses on various subjects by Drs. Blatchford, Brisbin, Bates, Thompson, Sprague, and Purple.

The following gentlemen are the officers for the present year:

Dr. Alexander H. Stevens, President.
" Alexander H. Thompson, Vice President.
" Peter Van Buren, Secretary.
" Peter Van Olinda, Treasurer.

